# Study on Average Housing Prices in the Inland Capital Cities of China by Night-time Light Remote Sensing and Official Statistics Data

**DOI:** 10.1038/s41598-020-64506-2

**Published:** 2020-05-07

**Authors:** Chang Li, Heli Zhu, Xinyue Ye, Chang Jiang, Jing Dong, Di Wang, Yijin Wu

**Affiliations:** 1 Key Laboratory for Geographical Process Analysing and Modelling, and College of Urban and Environmental Science, Central China Normal University, 152 Luoyu Road, Wuhan, 430079 China; 2 Department of informatics, New Jersey institute of technology, Newark, NJ USA

**Keywords:** Environmental economics, Socioeconomic scenarios

## Abstract

In this paper, the annually average Defense Meteorological Satellite Program-Operational Linescan System (DMSP/OLS) night-time light data is first proposed as a surrogate indicator to mine and forecast the average housing prices in the inland capital cities of China. First, based on the time-series analysis of individual cities, five regression models with gross error elimination are established between average night-time light intensity (ANLI) and average commercial residential housing price (ACRHP) adjusted by annual inflation rate or not from 2002 to 2013. Next, an optimal model is selected for predicting the ACRHPs in 2014 of these capital cities, and then verified by the interval estimation and corresponding official statistics. Finally, experimental results show that the quadratic polynomial regression is the optimal mining model for estimating the ACRHP without adjustments in most provincial capitals and the predicted ACRHP of these cities are almost in their interval estimations except for the overrated Chengdu and the underestimated Wuhan, while the adjusted ACRHP is all in prediction interval. Overall, this paper not only provides a novel insight into time-series ACRHP data mining based on time-series ANLI for capital city scale but also reveals the potentiality and mechanism of the comprehensive ANLI to characterize the complicated ACRHP. Besides, other factors influencing housing prices, such as the time-series lags of government policy, are tested and analysed in this paper.

## Introduction

The real estate industry is an important manifestation in the process of urbanization, and the housing price is a vital economic indicator reflecting the sustainability of regional development. Actually, the housing market has been particularly preoccupied late because of the excesses of rampant housing price growth, especially in Chinese cities. With the post-1978 reforms, China established a marketized system and shifted from a centrally-planned to a more market-based economy^1^ which means the market plays a dominant role in capital allocation and factor production^2^. A market-based system of housing provision was gradually founded since 1988 which promoted a vigorous urban housing market and caused housing prices skyrocketing^3^. As a barometer of national economic development, the soaring increasing housing prices in China’s cities has been concerned by many observers and analysts^4–6^. From 2005 to 2015, the price-income ratio is the nominal house price divided by the nominal disposable income per head (https://data.oecd.org/price/housing-prices.htm) of China’s leading real estate cities, Beijing and Shenzhen, increased from 7.69 to 13.37 and 5.95 to 15.54 by respectively calculating them from data sources on the websites: Shenzhen Municipal Statistics Bureau: http://www.sztj.gov.cn/; Beijing Municipal Bureau of Statistics: http://www.bjstats.gov.cn/tjsj/; National Bureau of Statistics of the People’s Republic of China: http://data.stats.gov.cn/search.htm. For housing markets, this rate is described as “affordable”, which is one of the key measures for a region’s socio-economy stability and should not exceed three times gross annual household income in general^7^. The ten-year trend of the rising housing price-income ratio shows that the price increases of commercial housing in China have been much higher than the increases in the ability of residents to pay. The implementation of the purchase restriction policy did not significantly affect housing prices but eased the impact of rising housing prices on technological innovation activities by suppressing excess. These cases demonstrate that housing prices in China should be studied urgently.

Chinese economic hypergrowth and urban ascent in the past 3 decades were driving forces behind the fast growth of housing markets in urban areas^8^. Likewise, the housing price is related to such factors as population migration and distribution, gross national product (GDP) and urbanization from a macro scale. For instance, housing price is tied with socio-economic components has been confirmed. A large number of economists pointed out the correlation between the GDP and housing prices^9,10^. The process of urbanization causing land price decrease directly to land use regulation restriction severely brings about increasing housing price^11,12^. And Regional variations in urbanization levels would affect housing prices^13^. In addition, population issues may also be related to housing prices, Saiz^14^ noted that immigration pushes up rents and housing values in US cities. Gonzalez and Ortega^15^ found that in the causal estimates of the effect of immigration on housing prices in Spain over the period 2000–2010, immigration was responsible for one-quarter of the increase in housing prices. Therefore, these indicators can be used to represent the development of regional housing prices.

Traditionally, the housing price data came from the census, which do not reflect timely market activity or the full scope of the regional estate market. However, it is worth noting that the night-time light imagery was used to estimate the influencing indicators of the housing price in real time and city scale. As a surrogate measure, the night-time light imagery has a potentiality to replace multiple indicators such as economic, social, resources and environmental circumstances^16–19^. For example, Elvidge *et al*.^20^ used the Defence Meteorological Satellite Program Operational Linescan System (DMSP-OLS) data to study the relationship between gross GDP, electric power consumption and light area in 21 countries and found that the light area was highly correlated to GDP and electric power consumption. Moreover, Doll *et al*.^21^ analysed night-time light remote sensing data of 11 European Union countries and the United States; and such data have been shown to correlate with national-level figures of GDP. Meanwhile, studies in China^22,23^, Africa^24^ and the United States^25^ have led to similar conclusions. Additionally, DMSP-OLS night-time light remote sensing data have been used in the study of urbanization and urban spatial expansion^26–29^, population migration and distribution^30–33^. Overall, previous studies have demonstrated that night-time light remote sensing data have been successfully used in social-economy factors, such as population migration and distribution, GDP, urbanization and so on.

Based on recursion that housing price is related to such factors as population migration and distribution, GDP and urbanization, and these factors can be predicted by night-time light remote sensing data, it can be deduced that there is a correlation between night-time light remote sensing data and housing price. The more frequent and dense human activities, the brighter light reflection and the more obvious result of night-time light remote sensing data. And the more frequent and dense human activities, the greater economic expansion and development. Night-time light remote sensing data and the socio-economic has a positive correlation, meaning they increase and decrease together^34^. The housing price is closely tied to these socio-economic factors. Therefore, the quantitative connections between night-time light remote sensing data and housing prices are robust and worth studying. In fact, there are few studies on the correlation between night-time light and the real estate market. E.g., Zhang^35^ estimated Chinese provincial real estate development time lags between land being purchased and the property being occupied using DMSP/OLS and real estate statistical data; and Wang *et al*.^36^ estimated Chinese housing vacancy rate using night-time light data and OpenStreetMap^37^ data. However, the aforementioned studies have researched many factors influencing housing prices instead of itself. Furthermore, owing to the spatial heterogeneity of China housing market, these researches indicated that using night-time lights alone for spatial modelling is insufficient to study housing markets. Therefore, this study provides a new perspective to mine the relationship between night-time light imagery and regional housing price using time-series analysis of individual cities, which avoids spatial differentiation of average housing prices at different cities. Meanwhile, there are some specific advantages for studying housing prices by night-time light data: Firstly, the night-time light date is objective. Night-time light date can directly reflect human activities to be used as a more objective data in socio-economic parameter estimation, most of which are emitted by human activities. In comparison to data in studying housing price in the field of economics, in the indicators for measuring economic development such as GDP are less objective and difficult to avoid statistical errors and human impact. Secondly, Night-time light data is easily available. It can be downloaded directly from the official website of the National Oceanic and Atmospheric Administration (NOAA). Compared with studying housing price researches in economics, they often require diversified indicator data for modelling, but these data are not easily available. Thirdly, the night-time light data has the advantages of dynamic updating and global coverage. With the existing relative background and the study of housing prices, we propose a regression model between annually average night-time light intensity (ANLI) and annually average commercial residential housing prices (ACRHP) for target provincial capital cities in inland China respectively. The work and contributions of this article are as follows:

(1) Based on the time-series analysis of individual cities, a new reliable data mining model between ANLI and ACRHP is first proposed. In order to guarantee that our study data are more reliable, we eliminated the abnormal errors of a few years and selecting an optimal mining model from several models to ensure the reliability of the results.

(2) The uncertainty of quantitative analysis about the prediction of ACRHP in the field of remote sensing is first studied and analysed by adjusting annual inflation rate or not. The traditional prediction usually obtains a certain value, whereas we propose a scientific and reasonable interval estimation to quantitatively measure the uncertainty of ACRHP using remote sensing.

(3) A new prediction method of night-time light intensity is proposed for the case of missing data for some years. The DMSP-OLS night-time light data are provided only until 2013; therefore, we propose a method for predicting intervals of night-time light intensity in subsequent years.

(4) Mining mechanism between ACRHP and ANLI is also first revealed. Moreover, the influence and lag of policy on ACRHP are also discussed by trend analysis.

(5) This paper not only enriches the application research of night-time light data but also provides a new reference point-of-view (i.e. using DMSP-OLS ANLI) to mining ACRHP in inland capital cities of China. It has great theoretical and practical significance for the real estate market.

## Study area and data

### Study area

**Figure 1:**
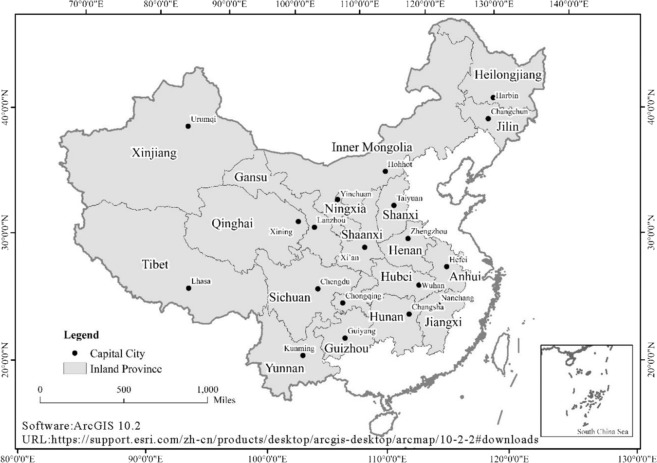
The capital cities of inland China.

The study area included 18 capital cities in inland China. They are Changchun, Changsha, Chengdu, Chongqing, Guiyang, Harbin, Hefei, Hohhot, Kunming, Lanzhou, Nanchang, Taiyuan, Urumqi, Wuhan, Xi’an, Xining, Yinchuan and Zhengzhou. Because of lacking official statistics of Lasha’s ACRHP, this paper doesn’t consider the inland capital city of Lasha. The locations of these cities are shown in Fig. 1. Comparing with the coastal city of China, these cities in inland China have appropriate urban estate economy developing scale and night-time light imagery quality.

Geographically, these inland capital cities cover most economy developing regions of China presently. All of these capital cities are important hub because they connect other parts of the province. Take Wuhan as an example, it’s a key role in China’s domestic transportation which has been regarded as the “thoroughfare to nine provinces”. With population agglomeration and urban expansion, the economy of Wuhan was developing rapidly in the past decade, representatively in estate economy. The local government took varied policy measures to stimulate the steady rise of housing prices, which provide a suitable condition for us to study housing prices.

In addition, comparing with the coastal region of China, the saturated digital number^38^ values of the light image in the economic status of the inland region before 2013 are not serious because of lagging in economic development. There only a few inland provincial capital cities have saturation problems close to 2013. Furthermore, this problem is only concentrated in part of these cities’ core area. The high degree of saturated DN values of light images may indeed have a certain impact on related research, but there is currently no approach recognized by the public to reduce the high degree of saturated DN values of light images. The existing methods mainly include radiation calibration, non-radioactive calibration and the vegetation adjusted night-time light to improve the saturation problem^39–41^. However, these methods have shortcomings and there is no officially recognized method. In this case, the accuracy of the desaturated data cannot be guaranteed. Therefore, choosing inland China as the study area is appropriate and ensuring the credibility of the results to a large extent.

### Study data

#### DMSP-OLS night-time light data

**Figure 2:**
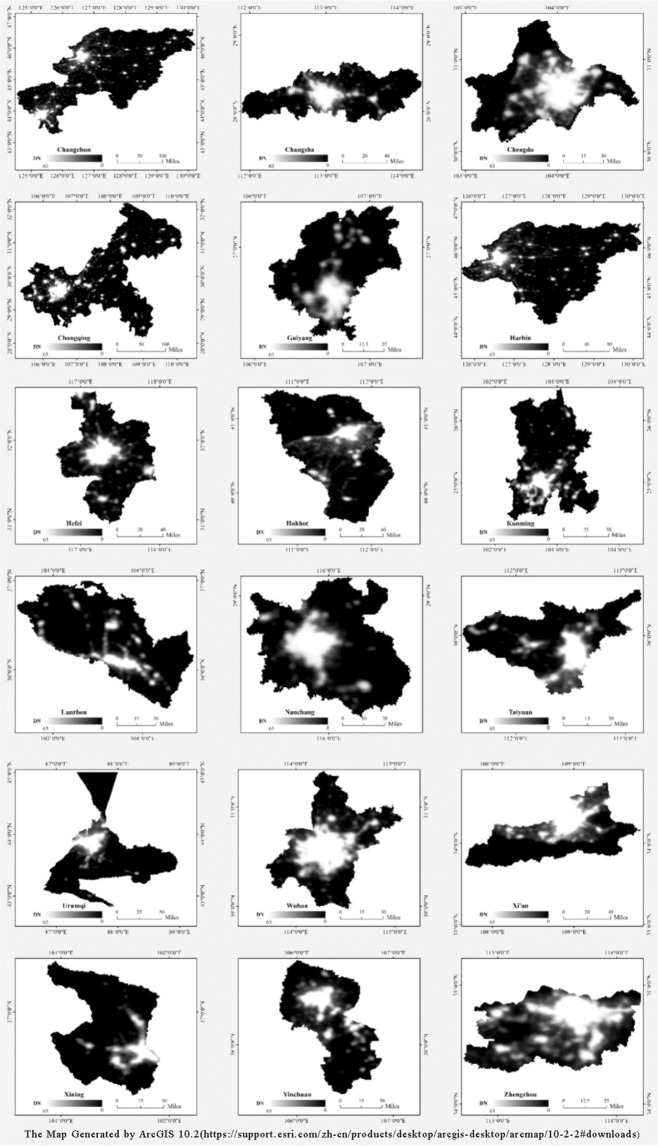
The 2013 DMSP-OLS data of the 18 inland provinces and provincial capitals in China.

In this article, we use the DMSP-OLS night-time light data to study housing prices. Comparing with NPP-VIIRS sensor launched in 2011 without history data and earth observation satellite Luojia 1-01 launched in mid-2018, the DMSP-OLS dataset can synthetize annual average data with long time-series historical data. The DMSP-OLS dataset was downloaded from the website of the NOAA (http://www.ngdc.noaa.gov/eog/dmsp/downloadV4composites.html). The data include average visible light, cloud-free coverage and stable light average data from 1992 to 2013. Accidental noise sources, such as clouds, lightning, flames and burning gases, have been eliminated in the stable light average dataset, which has values ranging from 1 to 63. We selected these datasets because some major outliers (such as those from fires) had already been discarded. Figure 2 shows the DMSP-OLS data for the 18 inland provinces and provincial capitals in China.

In this article, preprocessing of the DMSP-OLS data mainly included three steps:

(*a*) Reprojecting the imagery. To make it convenient to clip the imagery, the projection coordinate system was converted into the Lambert Conformal Conic system and the spheroid was converted into WGS 1984.

(*b*) Clipping the imagery. To make the imagery clearer, we clipped the DMSP-OLS stable light average data imagery and only kept the imagery of target cities.

(*c*) Intercalibrating radiometric information. To automatically extract the reference pixels with stable lights, the LMedS-based method^42^ was used to intercalibrate radiometric information.

#### Land area of administrative region data

To ensure that all the statistical data are unified and accurate, the land area data used in this paper are all from the China City Statistical Yearbook (2013). Table 1 shows the land area data of the 18 provincial capitals in China.

**Table 1:** The land area data of the administrative regions of the 18 provincial capitals.

City	Administrative area (square kilometre)	City	Administrative area (square kilometre)
Changchun	20565	Lanzhou	13100
Changsha	11819	Nanchang	7402
Chengdu	14335	Taiyuan	6988
Chongqing	82400	Urumqi	14216
Guiyang	8034	Wuhan	8494
Harbin	53100	Xi’an	10752
Hefei	11445	Xining	7679
Hohhot	17224	Yinchuan	9025
Kunming	21473	Zhengzhou	7446

#### Housing price data

To ensure that all the statistical data from 2002 to 2013 are unified and accurate, ACRHP data used in this paper are all from the China Statistical Yearbook (2002–2013). Table A (in the Appendix) shows the ACRHP data from 2002 to 2013 of 18 inland provincial capitals in China.

## Methodology

**Figure 3:**
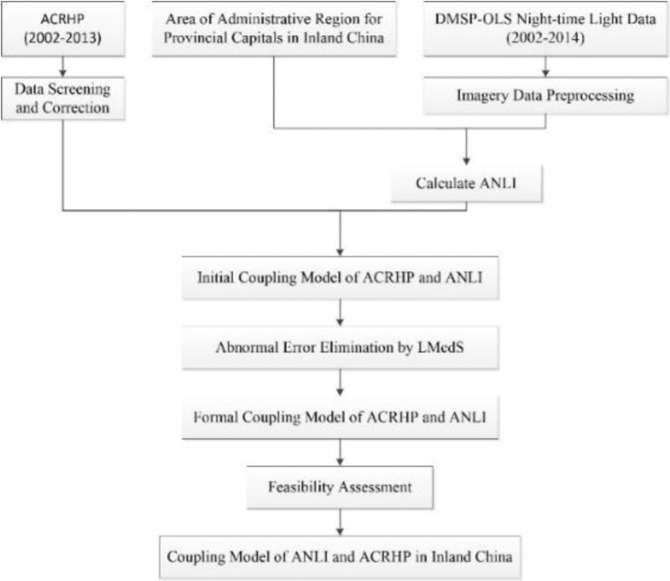
Flow chart of research processing.

In this study, we applied for cities’ polygon data from the National Geomatics Centre of China (http://ngcc.sbsm.gov.cn/). Then we overlaid the vector polygon data on the DMSP-OLS data and clipped out the target capital city imagery. After data preprocessed, the ANLI of each region is calculated and the correlation between annually ANLI and annually ACRHP is studied by establishing the regression model that is one of the important data mining methods. Next, we conduct a feasibility assessment to obtain the optimal mining model. Finally, we obtain and compare the results of the experiment. The process flow of our study is illustrated in Fig. 3.

### ACRHP adjusted by inflation rate

To correction variation of the data, the inflation rate was used to adjust the ACRHP. We obtained official inflation rate data form the World Bank (https://data.worldbank.org/). Table 2 shows Chinese annual inflation rate from 2002 to 2014. Table B (in the Appendix) shows the ACRHP data adjusted by the inflation index.

**Table 2:** Chinese inflation as measured by the annual growth rate of the GDP implicit deflator.

Year	2002	2003	2004	2005	2006	2007	2008	2009	2010	2011	2012	2013	2014
Rate (%)	0.60	2.61	6.95	3.90	3.93	7.75	7.79	−0.21	6.88	8.08	2.34	2.16	0.79

### Calculation of ANLI

First, given the problem of the inter-annual variation of night-time light, the exponential smoothing method was used in this study to obtain stable regional total night lights^43,44^. Then to calculate ANLI which represents the city night-time light intensity per unit of land area. It can be presented as a formula as follows:

1 $${\rm{ANLI}}=\mathop{\sum }\limits_{i=1}^{63}{N}_{i}{B}_{i}/S$$

In this formula, *N*_*i*_ represents the number of pixels with brightness *i*, *B*_*i*_ represents the brightness value itself, and *S* represents the land area data of the target capital city’s administrative region. Table 3 shows the ANLI calculation results from 2002 to 2013 of 18 provincial capitals in inland China.

**Table 3:** ANLI values of 18 provincial capitals in inland China (2002–2013).

City	ANLI											
	2002	2003	2004	2005	2006	2007	2008	2009	2010	2011	2012	2013
Changchun	5.757	6.828	9.148	7.856	7.214	8.715	10.638	12.724	17.819	14.340	11.478	13.064
Changsha	4.769	5.624	7.342	6.103	6.769	7.388	8.127	6.695	11.533	11.283	12.083	13.764
Chengdu	7.516	8.890	10.261	8.482	9.509	9.897	12.138	13.522	19.303	15.989	16.523	20.204
Chongqing	1.161	1.250	1.550	1.379	1.492	1.548	1.834	1.820	2.692	2.592	2.814	3.018
Guiyang	4.458	5.854	6.441	5.456	5.140	5.530	7.137	6.682	9.546	8.853	9.226	11.639
Harbin	3.734	3.874	6.038	4.416	4.267	4.583	6.196	8.167	8.679	6.886	7.420	8.302
Hefei	7.306	7.684	9.666	9.045	9.512	9.240	14.473	11.311	19.449	19.153	13.054	16.430
Hohhot	3.179	3.817	5.393	4.776	4.839	4.780	6.177	5.164	7.984	7.797	9.740	8.947
Kunming	3.944	3.973	4.544	3.777	4.183	4.467	5.890	5.863	9.428	8.165	9.394	9.312
Lanzhou	4.139	4.427	5.238	4.688	4.946	4.169	5.931	5.154	8.440	7.468	8.153	8.292
Nanchang	5.651	7.401	8.659	6.686	7.503	7.736	9.331	7.729	12.100	11.345	11.385	12.300
Taiyuan	11.039	11.672	14.207	12.361	12.398	10.996	13.938	12.371	17.887	15.892	17.103	16.903
Urumqi	6.196	6.288	6.713	6.338	7.689	6.932	6.598	7.696	10.796	10.852	11.097	12.233
Wuhan	11.596	12.027	14.858	12.748	13.984	15.241	18.389	14.174	23.903	22.813	22.806	27.090
Xi’an	8.673	8.842	11.049	10.170	10.968	10.175	13.471	13.229	19.151	16.582	18.079	19.169
Xining	3.314	3.611	4.493	3.913	4.046	3.890	5.353	5.811	8.289	7.114	7.448	7.400
Yinchuan	9.350	6.479	6.651	6.177	6.555	6.573	8.665	8.926	14.074	12.629	14.007	13.453
Zhengzhou	15.241	16.930	19.652	18.860	20.583	20.772	26.954	23.002	32.240	31.916	31.485	33.556

### Optimal regression model selection

Regression analysis is one of the classical statistical methods for data mining^45,46^, which can help to identify whether the correlation between two or more variables. In this study, the response variable is ACRHP and the explanatory variable is ANLI. Due to the spatial differentiation in Geographical science, the economic development levels of the provinces are usually different, and the ACRHP and ANLI data vary greatly. Hence, different empirical models are established for different cities in this paper, including the linear regression model, the exponential regression model, the logarithm regression model, the quadratic regression model, and the power regression model.

Linear regression model:

2 $${{\rm{ACRHP}}}_{j}=a{({\rm{ANLI}})}_{j}+b$$

Exponential regression model:

3 $${{\rm{ACRHP}}}_{j}=a{e}^{b{({\rm{ANLI}})}_{j}}$$

Logarithm regression model:

4 $${{\rm{ACRHP}}}_{j}=a\,{\log }_{b}{({\rm{ANLI}})}_{j}$$

Quadratic regression model:

5 $${{\rm{ACRHP}}}_{j}=a{{({\rm{ANLI}})}_{j}}^{2}+b{({\rm{ANLI}})}_{j}+c$$

Power regression model:

6 $${{\rm{ACRHP}}}_{j}=a({{\rm{ANLI}}}_{j}^{b})$$

where *a*, *b* and *c* are regression coefficients; *j* = 1, …, 18 refers to one capital city of observation. So, the optimal model for *j*-th city can be determined by:

7 $$\mathop{\max }\limits_{k=1,\mathrm{.}.,5}\{1-\sum _{i}{({{\rm{ACRHP}}}_{ik}-{\widehat{{\rm{ACRHP}}}}_{ik})}^{2}/\sum _{i}{({{\rm{ACRHP}}}_{ik}-{\overline{{\rm{ACRHP}}}}_{ik})}^{2}\}$$

where *k* means 1~5 different regression models corresponding Eq. (2)~(6) respectively; *i*=1,…,12 refers to year of observation at *j*-th capital city; $\widehat{{\rm{ACRHP}}}$ expresses an estimator by regression; and $\overline{{\rm{ACRHP}}}$ expresses a mean value.

We calculate the coefficient of determination (*R*^2^) of each existing regression and compare them to obtain the optimal model with the highest *R*^2^. It is worth noting that, statistically, the number of samples used in this experiment is sufficient. In this study, the essential observation number is 2 (because Eq. (2)~(6) usually includes 2 parameters *a* and *b*), and the observation number is 12 so that the degree of freedom (i.e. redundant observation) is 12–2 = 10. Hence redundant observation is sufficient.

### Abnormal error elimination

To prevent gross error (i.e. abnormal error) influences on the accuracy of the regression model between ANLI and ACRHP, least median of squares (LMedS)^42,47–49^ is used to eliminate gross errors (abnormal value). The objective function can be written:

8 $$\min \,[\mathop{{\rm{med}}}\limits_{i}({r}_{i}^{2})]$$

where ${r}_{i}$ is the *i*th residual error of the *i*th observation from Eq. (2)~(6). The “med” means the median. Then:

9 $${w}_{i}=\{\begin{array}{cc}1 & {\rm{if}}\,{{r}}_{i}^{2}\le {(2.5\times 1.4826(1+5/(n-l))\sqrt{{M}_{j}})}^{2}\\ 0 & {\rm{otherwise}}\end{array}$$

when *w*_*i*_ = 0, *M*_*j*_ is the minimal median for each subsample indexed by *J*; and *l* is the essential observation number of regression Eq. (2)~(6), which means 2.5-standard-deviation rule. Hence, outliers are removed by the LMedS.

After abnormal error elimination, regression models are again established and the *R*^2^ of each regression model is also calculated. By comparing the former *R*^2^ and the current *R*^2^ of each model, the regression model with the highest figure of *R*^2^ is selected to be the optimal mining model.

### Uncertainty estimation and performance evaluation

The ANLI of future years should be required in housing price prediction but the DMSP-OLS night-time light dataset was only updated to 2013. Considering that the night-time light data has the characteristics of being dynamic, stable and objective, we use time series prediction to avoid image distortion. The steps of housing price prediction are as follows:

(*a*) ANLI regression models for each provincial capital are established according to the time series; then, the function with the highest degree of *R*^2^ is selected as its regression model to predict the future ANLI of the target cities.

(*b*) The assumption of linear regression is used, and the nonlinear function of ANLI prediction should be transformed into the linear function: ${Y}_{0}={b}_{0}+{b}_{1}{x}_{0}$. Combined with the target cities, the predicted function is assumed to be:

10 $${\hat{Y}}_{0}={\hat{b}}_{0}+{\hat{b}}_{1}{x}_{0}$$

where ${\hat{b}}_{1}$ and ${\hat{b}}_{0}$ represent coefficients of the linear function predicting ANLI for target cities by parameter estimation.

The interval estimation of ${Y}_{0}$ is as follows:

11 $$({\hat{Y}}_{0}\pm {t}_{\alpha /2}\,(n-2)\hat{\sigma }\sqrt{1+\frac{1}{n}+\frac{{({x}_{0}-\bar{x})}^{2}}{{\sum }_{i=1}^{n}{({x}_{i}-\bar{x})}^{2}}})$$

where *n* represents sample size, $\hat{{\rm{\sigma }}}$ represents population standard deviation, $\bar{x}$ represents sample mean, ${t}_{\alpha /2}$ represents a value of confidence level (α) corresponding to *T*-distribution, and $\alpha =0.05$.

(*c*) ANLI interval estimation for target cities of future years is calculated by MATLAB (the software package).

To ensure the authenticity of the model, the optimal data mining model should be a progressive feasibility assessment. The ANLI interval estimation of future years is used in the optimal data mining model between annually ACRHP and annually ANLI; therefore, the result of the ACRHP interval estimation is calculated. Finally, we compare this result with the official statistical ACRHP published by the National Bureau of Statistics of the target cities to demonstrate feasibility. Therefore, the optimal data mining model is verified and can be used to predict housing prices.

## Experimental results and analysis

### The coupling results of ANLI and ACRHP

Hefei is taken as an example and five regression models between ANLI and ACRHP are established so that the optimal regression model can be obtained by comparing the *R*^2^. Table 4 shows five regression models between ANLI and ACRHP for Hefei.

**Table 4:** All the regression models of ANLI and ACRHP for Hefei.

Model	Formula	*R*^2^
Power regression model	ACRHP = 264.6ANLI^1.07^	0.8730
Linear regression model	ACRHP = 343.8ANLI − 333.9	0.8744
Quadratic regression model	ACRHP = −10.36ANLI^2^ + 623.2ANLI − 2009	0.8831
Exponential regression model	ACRHP = 1382e^0.07934ANLI^	0.8468
Logarithm regression model	ACRHP = 4372ln(ANLI) − 6818	0.8823

(*R*^2^ represents the coefficient of determination used to evaluate the accuracy and reasonableness of the coupling models.)

After comparing all the above-mentioned regression models, including the linear regression model, the exponential regression model, the logarithm regression model, the quadratic regression model, and the power regression model, we conclude that the regression model with the highest figure of *R*^2^ is the Quadratic regression model (88.95%). Therefore, we can approximately conclude that the Quadratic regression model is the optimal mining model for predicting housing prices.

The regression models of the other 17 target cities are calculated in the same way. And Table 5 shows five regression models for Changchun, Changsha, Chengdu, Chongqing, Guiyang, Harbin, Hohhot, Kunming, Lanzhou, Nanchang, Taiyuan, Urumqi, Wuhan, Xi’an, Xining, Yinchuan, and Zhengzhou.

**Table 5:** All regression models for the other 17 cities.

City	Linear regression model	*R*^2^
Changchun	ACRHP = 490.3ANLI − 1134	0.7991
Changsha	ACRHP = 516.8ANLI − 630.9	0.9100
Chengdu	ACRHP = 456.3ANLI − 1091	0.8642
Chongqing	ACRHP = 2099ANLI − 887.4	0.9510
Guiyang	ACRHP = 628ANLI − 1036	0.8661
Harbin	ACRHP = 760.3ANLI − 644.1	0.8289
Hohhot	ACRHP = 654.9ANLI − 959.7	0.9297
Kunming	ACRHP = 590.7ANLI + 175.5	0.9639
Lanzhou	ACRHP = 756.5ANLI − 1040	0.8096
Nanchang	ACRHP = 789ANLI − 3007	0.8718
Taiyuan	ACRHP = 661.2ANLI − 4331	0.8814
Urumqi	ACRHP = 618.1ANLI − 1601	0.9032
Wuhan	ACRHP = 364.6ANLI-1962	0.9146
Xi’an	ACRHP = 462ANLI − 1927	0.9598
Xining	ACRHP = 701.6ANLI − 874.6	0.9135
Yinchuan	ACRHP = 322.2ANLI + 184.6	0.8540
Zhengzhou	ACRHP = 248.6ANLI − 2069	0.8858
**City**	**Quadratic regression model**	***R***^**2**^
Changchun	ACRHP = 43.53ANLI^2^ − 384.7ANLI + 2942	0.8316
Changsha	ACRHP = −9.176ANLI^2^ + 686ANLI − 1329	0.9113
Chengdu	ACRHP = −22.84ANLI^2^ + 1076ANLI − 4904	0.8906
Chongqing	ACRHP = −66.95ANLI^2^ + 2379ANLI − 1152	0.9512
Guiyang	ACRHP = −38.14ANLI^2^ + 1173ANLI − 2865	0.8710
Harbin	ACRHP = 31.16ANLI^2^ + 380.2ANLI + 422.1	0.8312
Hohhot	ACRHP = 48.33ANLI^2^ + 26.62ANLI + 868.4	0.9446
Kunming	ACRHP = −0.8514ANLI^2^ + 601.9ANLI + 142.8	0.9640
Lanzhou	ACRHP = 2.221ANLI^2^ + 728.4ANLI − 956.9	0.8097
Nanchang	ACRHP = 81.99ANLI^2^ − 713.4ANLI + 3516	0.9021
Taiyuan	ACRHP = 5.554ANLI^2^ + 501.6ANLI − 3219	0.8816
Urumqi	ACRHP = 61.19ANLI^2^ − 483ANLI + 3035	0.9151
Wuhan	ACRHP = −12.92ANLI^2^ + 852.6ANLI − 6196	0.9327
Xi’an	ACRHP = 5.921ANLI^2^ + 298ANLI − 872.7	0.9610
Xining	ACRHP = 98.83ANLI^2^ − 381.6ANLI + 1855	0.9304
Yinchuan	ACRHP = 25.34ANLI^2^ − 183.8ANLI + 2481	0.8724
Zhengzhou	ACRHP = 3.981ANLI^2^ + 49.85 ANLI + 250.7	0.8899
**City**	**Logarithm regression model**	***R***^**2**^
Changchun	ACRHP = 4519ln (ANLI) − 6465	0.7454
Changsha	ACRHP = 4448ln (ANLI) − 5504	0.9033
Chengdu	ACRHP = 5873ln (ANLI) − 9938	0.8836
Chongqing	ACRHP = 4139ln (ANLI) + 658.6	0.9429
Guiyang	ACRHP = 4331ln (ANLI) − 4927	0.8682
Harbin	ACRHP = 4359ln (ANLI) − 3708	0.8068
Hohhot	ACRHP = 3816ln (ANLI) − 3643	0.8713
Kunming	ACRHP = 3646ln (ANLI) − 2591	0.9547
Lanzhou	ACRHP = 4608ln (ANLI) − 4592	0.8022
Nanchang	ACRHP = 6782ln (ANLI) − 10630	0.8293
Taiyuan	ACRHP = 9328ln (ANLI) − 19540	0.8768
Urumqi	ACRHP = 5344ln (ANLI) − 7609	0.8850
Wuhan	ACRHP = 6613ln (ANLI) − 14230	0.9299
Xi’an	ACRHP = 6065ln (ANLI) − 11260	0.9416
Xining	ACRHP = 3620ln (ANLI) − 3042	0.8841
Yinchuan	ACRHP = 3001ln (ANLI) −3368	0.8195
Zhengzhou	ACRHP = 5909ln (ANLI) − 14680	0.8674
**City**	**Exponential regression model**	***R***^**2**^
Changchun	ACRHP = 923.8e^0.134ANLI^	0.8257
Changsha	ACRHP = 1232e^0.1239ANLI^	0.8854
Chengdu	ACRHP = 1532e^0.08369ANLI^	0.7966
Chongqing	ACRHP = 908.2e^0.6073ANLI^	0.9357
Guiyang	ACRHP = 911.2e^0.1792ANLI^	0.8406
Harbin	ACRHP = 1183e^0.1906ANLI^	0.8233
Hohhot	ACRHP = 806.5e^0.2028ANLI^	0.9395
Kunming	ACRHP = 1455e^0.1476ANLI^	0.9560
Lanzhou	ACRHP = 969.4e^0.2045ANLI^	0.8019
Nanchang	ACRHP = 643.5e^0.1961ANLI^	0.9047
Taiyuan	ACRHP = 765.6e^0.1292ANLI^	0.8739
Urumqi	ACRHP = 857.5e^0.1625ANLI^	0.9137
Wuhan	ACRHP = 1194e^0.07092ANLI^	0.8573
Xi’an	ACRHP = 979.4e ^0.1042ANLI^	0.9493
Xining	ACRHP = 711.6e^0.2474ANLI^	0.9317
Yinchuan	ACRHP = 1229e^0.09771ANLI^	0.8688
Zhengzhou	ACRHP = 816.5e^0.06194ANLI^	0.8893
**City**	**Power regression model**	***R***^**2**^
Changchun	ACRHP = 154.2ANLI^1.379^	0.8116
Changsha	ACRHP = 313.4ANLI^1.157^	0.9084
Chengdu	ACRHP = 225.6ANLI^1.191^	0.8557
Chongqing	ACRHP = 1347ANLI^1.272^	0.9495
Guiyang	ACRHP = 264.2ANLI^1.301^	0.8620
Harbin	ACRHP = 466.2ANLI^1.183^	0.8306
Hohhot	ACRHP = 251ANLI^1.361^	0.9392
Kunming	ACRHP = 676.3ANLI^0.953^	0.9639
Lanzhou	ACRHP = 338.7ANLI^1.296^	0.8096
Nanchang	ACRHP = 69.76ANLI^1.834^	0.8922
Taiyuan	ACRHP = 34ANLI^1.876^	0.8806
Urumqi	ACRHP = 159.1ANLI^1.454^	0.9085
Wuhan	ACRHP = 81.29ANLI^1.389^	0.9020
Xi’an	ACRHP = 91.67ANLI^1.47^	0.9609
Xining	ACRHP = 295.3ANLI^1.346^	0.9200
Yinchuan	ACRHP = 378.1ANLI^0.9556^	0.8527
Zhengzhou	ACRHP = 27.08ANLI^1.554^	0.8893

Observing all the experimental results, we can conclude that the optimal mining model for Changchun, Changsha, Chengdu, Chongqing, Guiyang, Harbin, Hefei, Hohhot, Kunming, Lanzhou, Taiyuan, Wuhan, Xi’an, Yinchuan, Urumqi, and Zhengzhou is the quadratic regression model, while the optimal mining method for Nanchang and Xining is the exponential regression model.

### Abnormal error elimination and optimal model determination

**Figure 4:**
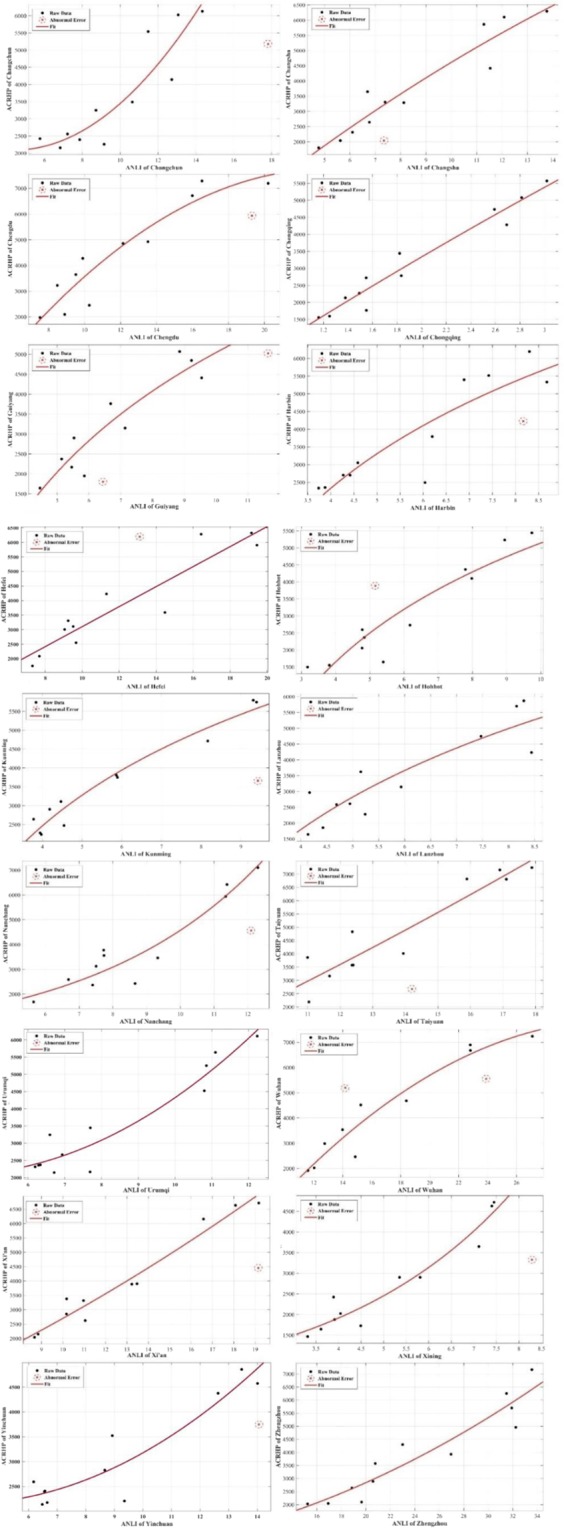
The abnormal errors and curve fittings for each capital city.

Figure 4 shows the curve fittings and the abnormal errors of each capital city. The abnormal errors of the optimal model of each capital city are eliminated by the LMedS algorithm.

Table 6 shows the results of again establishing the regression model after eliminating the abnormal errors.

**Table 6:** The optional regression models of ANLI and ACRHP after eliminating the abnormal errors.

City	Optional Regression Model	*R*^2^
Changchun	ACRHP = 43.53ANLI^2^ − 384.7ANLI + 2942	0.8316
Changsha	ACRHP = −9.176ANLI^2^ + 686ANLI − 1329	0.9113
Chengdu	ACRHP = −22.84ANLI^2^ + 1076ANLI − 4904	0.8906
Chongqing	ACRHP = −66.95ANLI^2^ + 2379ANLI − 1152	0.9512
Guiyang	ACRHP = −38.14ANLI^2^ + 1173ANLI − 2865	0.8710
Harbin	ACRHP = 31.16ANLI^2^ + 380.2ANLI + 422.1	0.8312
Hefei	ACRHP = −10.36ANLI^2^ + 623.2ANLI − 2009	0.8831
Hohhot	ACRHP = 48.33ANLI^2^ + 26.62ANLI + 868.4	0.9446
Kunming	ACRHP = −0.8514ANLI^2^ + 601.9ANLI + 142.8	0.9640
Lanzhou	ACRHP = 2.221ANLI^2^ + 728.4ANLI − 956.9	0.8097
Nanchang	ACRHP = 643.5e^0.1961 ANLI^	0.9047
Taiyuan	ACRHP=5.554ANLI^2^ + 501.6ANLI − 3219	0.8816
Urumqi	ACRHP = 61.19ANLI2-483ANLI + 3035	0.9151
Wuhan	ACRHP = −12.92ANLI^2^ + 852.6ANLI − 6196	0.9327
Xi’an	ACRHP = 5.921ANLI^2^ + 298ANLI − 872.7	0.9610
Xining	ACRHP = 711.6e^0.2474ANLI^	0.9317
Yinchuan	ACRHP = 25.34ANLI^2^−183.8ANLI + 2481	0.8724
Zhengzhou	ACRHP = 3.981ANLI^2^ + 49.85 ANLI + 250.7	0.8899

Abnormal error elimination can significantly improve the accuracy of the mining model. To reduce the impact of the abnormal error on the accuracy of the mining model, the abnormal error of ANLI and ACRHP are eliminated after obtaining the optimal model. Comparing the current regression models with the former regression models (Table 5, Table 6), the accuracies of the models are significantly improved. The results of comparing the two situations of the same city’s regression models that eliminate abnormal error show that the optimal mining relationship between ACRHP and ANLI for Changchun, Changsha, Chengdu, Chongqing, Guiyang, Harbin, Hefei, Hohhot, Kunming, Lanzhou, Taiyuan, Wuhan, Xi’an, Yinchuan, Urumqi, and Zhengzhou is the quadratic function, while for Nanchang and Xining is the exponential regression model.

### Uncertainty estimation of prediction

#### Predicted future housing prices

ANLI regression models of each provincial capital according to their time series are established. The explained variable is ANLI and the explanatory variable is year *Y*. The calculation is based on the ANLI of the previous time series, and the function with the highest degree of *R*^2^ is selected as its regression model. The optimal regression model of the ANLI time series prediction of each provincial capital is shown in Table 7.

**Table 7:** The optimal regression model for the ANLI time series prediction of each provincial capital.

City	Regression model	*R*^2^
Changchun	ANLI = −0.01829*Y*^2^ + 74.13*Y* − 75090	0.7997
Changsha	ANLI = 0.06177*Y*^2^ − 247.2*Y* + 247400	0.8891
Chengdu	ANLI = 0.06861*Y*^2^ − 274.4*Y* + 274300	0.8672
Chongqing	ANLI = 0.01298*Y*^2^ − 51.95*Y* + 51970	0.9354
Guiyang	ANLI = 0.05905*Y*^2^ − 236.6*Y* + 236900	0.8707
Harbin	ANLI = −0.0007979*Y*^2^ + 3.633*Y* − 4071	0.7085
Hefei	ANLI = 0.01278*Y*^2^ − 50.19*Y* + 49270	0.7457
Hohhot	ANLI = 0.0321*Y*^2^ − 128.3*Y* + 128300	0.8707
Kunming	ANLI = 0.04935*Y*^2^ − 197.5*Y* + 197700	0.8766
Lanzhou	ANLI = 0.0353*Y*^2^ − 141.3*Y* + 141400	0.8032
Nanchang	ANLI = 0.0296*Y*^2^ − 118.3*Y* + 118200	0.7849
Taiyuan	ANLI = 0.04523*Y*^2^ − 181.1*Y* + 181200	0.6653
Urumqi	ANLI = 0.06364*Y*^2^ − 254.9*Y* + 255300	0.8960
Wuhan	ANLI = 0.1048*Y*^2^ − 419.3*Y* + 419600	0.8644
Xi’an	ANLI = 0.051*Y*^2^ − 203.7*Y* + 203500	0.8800
Xining	ANLI = 0.01513*Y*^2^ − 60.31*Y* + 60100	0.8229
Yinchuan	ANLI = 24.81*Y*^2^ − 99330*Y* + 99440000	0.9650
Zhengzhou	ANLI = 0.03563*Y*^2^ − 141.3*Y* + 140100	0.9074

**Figure 5:**
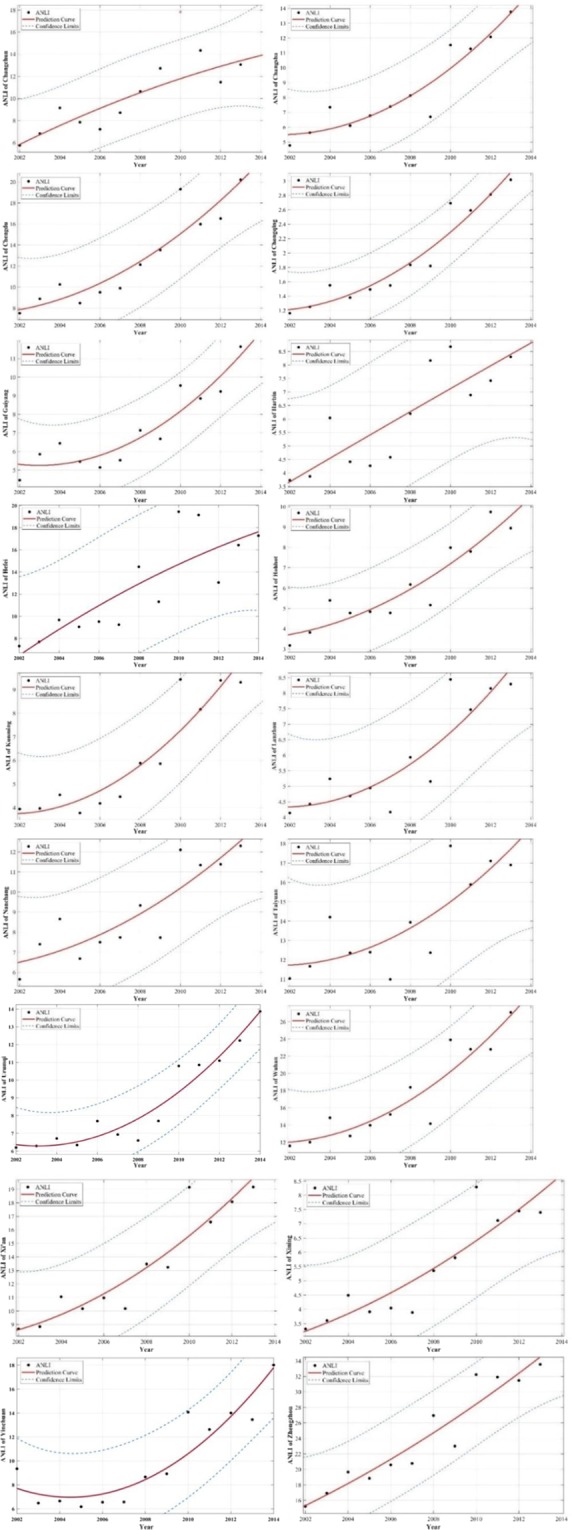
Prediction of ANLI values of cities in 18 inland provinces (The ANLI Time Series of the capital city and its 95% Confidence Interval).

Using the principle of least-squares curve fitting for regression analysis and prediction, the ANLI of the 18 provincial capitals in future years can be obtained. Figure 5 shows the results of taking 2014 as an example to evaluate the rationality of each city’s model and predict the future ANLI. Table 8 lists the ANLI prediction intervals for each capital city.

**Table 8:** ANLI prediction interval for each capital city in 2014.

City	Average luminous intensity prediction interval	City	Average luminous intensity prediction interval
Changchun	[3693.2201, 9282.6263]	Lanzhou	[4776.6523, 7761.3826]
Changsha	[5873.4784, 7930.1599]	Nanchang	[5284.7410, 15338.0345]
Chengdu	[7019.7011, 7614.2461]	Taiyuan	[5618.3684, 10893.5513]
Chongqing	[5412.0118, 7084.2757]	Urumqi	[6030.3194, 10613.1091]
Guiyang	[5210.2818, 6121.5320]	Wuhan	[6885.5624, 7843.6955]
Harbin	[4012.9208, 8780.8144]	Xi’an	[6373.7022, 10303.8156]
Hefei	[3772.4103, 13068.3891]	Xining	[3828.2856, 9908.7087]
Hohhot	[4617.5183, 8652.1139]	Yinchuan	[4427.4706, 5773.0676]
Kunming	[5650.3894, 8119.2782]	Zhengzhou	[5765.1616, 9320.3745]

The obtained ANLI prediction interval is brought into the optimal regression model of ANLI and AHP of each capital city, and the housing price range of 2014 can be calculated and finally compared with the price published by the National Bureau of Statistics to test the model. Taking Hefei as an example, the data show that ANLI_MIN_ = 14.0021 and ANLI_MAX_ = 22.5585. The possible average housing price prediction interval is: ACRHP_MIN_ = 5640.0554 yuan per square metre and ACRHP_MAX_ = 8606.2131 yuan per square metre. The housing price of Hefei from the 2014 official statistics is 7157 yuan per square metre, which is within this prediction interval. Table 9 shows the ACRPH prediction range and actual housing price for each provincial capital.

**Table 9:** ACRPH prediction range and actual housing price for each provincial capital.

City	ACRHP prediction range (yuan per square metre)	Actual Housing Price (yuan per square metre)
Changchun	[3693.2201, 9282.6263]	6261
Changsha	[5873.4784, 7930.1599]	6116
Chengdu	[7019.7011, 7614.2461]	7032
Chongqing	[5412.0118, 7084.2757]	5519
Guiyang	[5210.2818, 6121.5320]	5608
Harbin	[4012.9208, 8780.8144]	6182
Hefei	[3772.4103, 13068.3891]	7157
Hohhot	[4617.5183, 8652.1139]	5474
Kunming	[5650.3894, 8119.2782]	6384
Lanzhou	[4776.6523, 7761.3826]	6460
Nanchang	[5284.7410, 15338.0345]	6589
Taiyuan	[5618.3684, 10893.5513]	7651
Urumqi	[6030.3194, 10613.1091]	6429
Wuhan	[6885.5624, 7843.6955]	7951
Xi’an	[6373.7022, 10303.8156]	6465
Xining	[3828.2856, 9908.7087]	5753
Yinchuan	[4427.4706, 5773.0676]	4451
Zhengzhou	[5765.1616, 9320.3745]	7571

From the results above, the prediction results are mainly accurate. As seen in Table 9, one unanticipated finding was that the ACRHP of Chengdu was overestimated 88 yuan, while the Wuhan was underestimated 107 yuan.

#### Optimization prediction results

As the results above, the uncertainty of ANLI is considered, while the uncertainty of ACRHP is ignored. To improve the accuracy of our optimal model, the ACRHP was adjusted by official inflation rate data acquiring form the World Bank.

Table 10 shows ACRPH prediction range and actual housing price for each provincial capital after ACRHP corrected by Chinese inflation rate. From the results above, the prediction results are all in our prediction interval which further confirms the feasibility and accuracy of our method.

**Table 10:** ACRPH prediction range and actual housing price for each provincial capital after ACRHP correction.

City	ACRHP prediction range (yuan per square metre)	Actual Housing Price (yuan per square metre)
Changchun	[3544.5635, 8888.9669]	6261
Changsha	[5649.8158, 8248.2110]	6116
Chengdu	[6778.8102, 7852.3651]	7032
Chongqing	[5249.2187, 7200.5541]	5519
Guiyang	[4915.3325, 5678.2948]	5608
Harbin	[3770.5184, 8801.3779]	6182
Hefei	[3742.8418, 12965.9581]	7157
Hohhot	[4415.4099, 8824.4203]	5474
Kunming	[5484.6389, 8472.8164]	6384
Lanzhou	[4889.5986,12456.7310]	6460
Nanchang	[4313.1572,10888.5346]	6589
Taiyuan	[5370.5871,10396.7109]	7651
Urumqi	[5983.0532, 10529.9227]	6429
Wuhan	[7024.0785, 9192.6769]	7951
Xi’an	[6154.3089,10481.8340]	6465
Xining	[3690.1516, 9673.1074]	5753
Yinchuan	[4392.7677, 5727.8178]	4451
Zhengzhou	[5489.3769,10420.6243]	7571

### Discussion

#### The experimental sample size

According to the principle of statistical inference, when a small probability event occurs, it cannot be considered as an accident event. We selected *m* = 18 inland provincial capital cities in China as test areas. There are two outcomes for predicting ACRHP, consistency or inconsistency. For *m* provincial capital cities, there are 2^*m*^ cases (R^2^ is high or low, namely consistency or inconsistency). Two sets of experiments were undertaken to compare the performance.

The first set of experiments forecasted ACRHP by ANLI directly. There are two cities out of prediction interval, i.e., Chengdu and Wuhan. Therefore, the probability of the strong correlation between ACRHP and ANLI of all the *m* provincial capitals is ${C}_{18}^{2}/{2}^{18}=153/262144=0.0005836487,$ which is a very small probability event. The second set of experiments used the adjusted ACRHP by inflation index for prediction. All cities are in the prediction interval. The probability of the strong correlation between ACRHP and ANLI of all the *m* provincial capitals is only $1/{2}^{18}=1/262144=0.0000038147$, which is the much smaller probability event.

All in all, the experimental results verify that our sample size is enough, scientific and reliable; so there is a strong satistical correlation relationship between ACRHP and ANLI for 18 inland provincial capital cities in China.

#### The influence of saturation problem

The saturation problem of DMSP/OLS data has little effect in this research. The reason is as follows.

(*a*) For this study, there only few inland provincial capital cities in China have saturation problems close to 2013. Moreover, this problem is only concentrated in certain few areas of the developed city centre such as Wuhan and Chengdu.

(*b*) Research object is the city-scale ACRHP, so “average” night-time light intensity (ANLI) is used to analyse housing prices which can smooth (i.e. “average”) or decrease the saturated error of night-time light brightness value. In other words, the error, which exceeds 63, divided by the very large *S* is almost ignored. *S* represents an administrative area of provincial capital city in Eq. (1), and provincial capital cities are always with the large areas, e.g., the smallest inland provincial capital city - Taiyuan is 6988 km^2^.

(*c*) Saturation processing of DMSP-OLS may introduce new errors due to spatial heterogeneity. Therefore, we selected inland China as study areas where the saturation problem is not serious to ensure the credibility of the results to a large extent.

#### The mechanism between ACRHP and ANLI

It can be seen from the experimental results that the correlation degrees of the ANLI and ACRHP for the 18 provincial capitals in inland China are satisfactory. The optimal mining model is the quadratic regression model. In addition, ACRHP can be used to predict the future ACRHP. The relevant information can be summarized as follows.

(*a*) ANLI and ACRHP are highly correlated. Firstly, the correlation between ANLI and ACRHP can be explained by the internal mechanism. As mentioned in the Introduction section, there is a transmission mechanism between night-time light and ACRHP. Housing price is related to such social-economy factors as population migration and distribution, gross national product (GDP), and urbanization from a macro point of view. And these social-economy factors can be reflected and represented from night-time light imagery. In all, this conduction effect can be generalized by the substitution of the representation Eq. (12). Secondly, the experimental results strongly demonstrate that there is indeed a strong correlation between ANLI and ACRHP. In the process of constructing regression models of ANLI and ACRHP, as shown in Table 6, the ${R}^{2}$ of each regression model is above 0.80, which demonstrates that there is a high correlation between ANLI and ACRHP.

12 $$\begin{array}{c}ACRHP=f({x}_{1},{x}_{2},\cdots ,{x}_{i})\\ {x}_{1}={g}_{1}(NTL)\\ {x}_{2}={g}_{2}(NTL)\\ \cdots \cdots \\ {x}_{n}={g}_{i}(NTL)\\ ACRHP=f({g}_{1}(NTL),{g}_{2}(NTL),\cdots ,{g}_{i}(NTL))={\rm{F}}(NTL)\end{array}$$

where *f*(*x*) and *g*(*x*) represent the functional relationship; ${x}_{i}$ represents social-economy factors such as population, gross national product, human activities, urbanization and so on; $g({\rm{NTL}})$ represents the quantity relationship between these social-economy factors and brightness value of night-time light imagery. Based on recursion, we can acquire a composite function ─ $ACRHP=F(NTL)$ which reflects the transmission mechanism between NTL and ACRHP.

(*b*) Overall, the optimal mining model between ANLI and ACRHP of the most inland provincial capitals in China is the quadratic function, which can be regarded as an empirical formula. Additionally, the optimal mining model is quadratic function can be explained by Taylor series. In mathematics, a Taylor series is a representation of a function as an infinite sum of terms that are calculated from the values of the function’s derivatives at a single point. The Taylor series of a real or complex-valued function *f (x)* that is infinitely differentiable at a real or complex number a is the power series

13 $$f(a)+\frac{f{\prime} (a)}{1!}(x-a)+\frac{f{\prime\prime} (a)}{2!}{(x-a)}^{2}+\frac{f\prime\prime\prime (a)}{3!}{(x-a)}^{3}+\ldots $$

which can be written in the more compact sigma notation as

14 $$\mathop{\sum }\limits_{n=0}^{\infty }\frac{{f}^{(n)}(a)}{n!}{(x-a)}^{n}$$

where *n*! denotes the factorial of *n* and *f*^(*n*)^(*a*) denotes the *n*th derivative of *f* evaluated at the point *a*. Any elementary function can be approximated by using a finite number of terms of its Taylor series. The optimal mining model is the quadratic polynomial, which can approximate any arbitrary function relationship. For this reason, the quadratic polynomial can be used to explain the relationship between ANLI and ACRHP more accurately. However, the quadratic function is only an optimal model in the capital cities of the most provinces in inland China, and it is an empirical model. Due to spatial differentiation, different cities may have different optimal models.

(*c*) ANLI can be used to predict the future ACRHP of provincial capitals in China. Based on the conclusion that ANLI and ACRHP are highly correlated, we predict the ACRHP in the following years of the target cities and compare them with the data published by the National Bureau of Statistics, with satisfactory results (Table 9). Among the results, the actual housing price of Chengdu and Wuhan in 2014 slightly deviates from the predicted housing price. Obviously, the 2014 DMSP-OLS night-time light intensity is calculated by establishing a regression curve of the time series prediction, which may make the 2014 night-time light intensity itself uncertain: when it is used to predict the housing price, it may lead to some deviation. However, this “unusual case” also can be reasonably explained by socio-economic factors.

For Chengdu, the ACRHP is overestimated 88 yuan. There are several possible explanations for this result. Firstly, natural disasters may influence the purchase behaviours, especially the earthquake, which usually causes a temporary real estate marketing crisis because of the negative consequences affecting the buildings^50^. Prior studies have noted that the 2008 Wenchuan earthquake (the deadliest earthquake to hit China in the past three decades) changed the consumption concept and the consumption behaviour of the resident^51^. According to official statistics, the ACRHP of Chengdu is 4778 yuan in 2008, while the ACRHP of Wuhan is 4781 yuan. By 2016, the ACRHP in Chengdu has increased to 7504 yuan, but the ACRHP in Wuhan has exceeded 10,000 yuan. In addition, the urban planning by local government is another important factor caused low ACRHP in Chengdu. In 2006, the government set a goal to construct a high-density city which improved floor area ratio and reduced the cost of real estate developers. All in all, the natural disasters and land policies have jointly led to moderate growth of the ACRHP in Chengdu.

For Wuhan, the ACRHP is underestimated 107 yuan. This finding was unexpected and suggests that the size of its economies and the change of corresponding policies may be the main factor. Table 11 shows the GDP of each capital city and its rank among all Chinese cities in 2014. Wuhan has a high economic level with its GDP in 2014 ranked eighth in cities across China, and the saturated digital number^38^ values of the light image is serious problem. Furthermore, in 2014, Wuhan abolished housing purchase restriction began in 2012 which brought “real estate market heat” and boosted the sale of houses. Therefore, the housing price of Wuhan experienced a big rise in 2014.

**Table 11:** The GDP of each capital city and its rank among all Chinese cities in 2014.

City	GDP in 2014 (100 million yuan)	Rank among all Chinese cities
Chongqing	14265	6
Wuhan	10069	8
Chengdu	10057	9
Changsha	7825	14
Zhengzhou	6783	19
Xi’an	5475	26
Changchun	5382	27
Harbin	5333	28
Hefei	5158	30
Kunming	3713	42
Nanchang	3668	44
Hohhot	2894	63
Taiyuan	2413	72
Urumqi	2510	75
Guiyang	2497	77
Lanzhou	1905	97
Yinchuan	1273	139
Xining	979	193

In addition to the above possible reasons, the official statistical housing prices of Chengdu and Wuhan are slightly deviates from the predicted housing price, and the actual values of the other 16 cities are accurately in the prediction range. Therefore, the average DMSP-OLS night-time light intensity can be used to predict the future ACRHP.

Besides, the reasons why the policy factors only have a slight impact on our regression model in most inland capital cities can be explained as below:

Firstly, the direct government intervention cannot radically change the driving mechanism of the housing prices especially in a market economy^52^. Meanwhile, indirect intervention already reflected in night-time lighting. For example, the Chinese government reformed the hukou system to adapt the current trend that populations are diverse moving from rural to urban centres, which promotes urbanization in China. And the series anthropogenic factors change already reflected in night-time light imagery.

**Figure 6:**
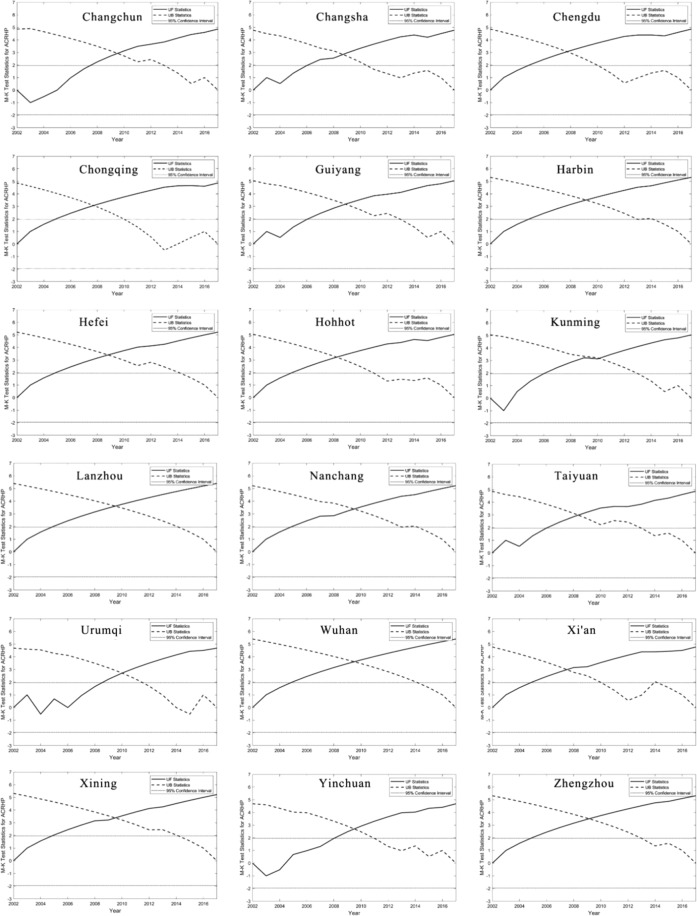
Mann-Kendall trend test of ACRHP at 18 inland capital cities in China during 2002–2017 (UF > 0 represents an increasing trend, while UF < 0 represents a decreasing trend. And if UF beyond 95% confidence interval line represents the increasing trend or decreasing trend is significant).

Secondly, the available DMSP-OLS data until 2013 when the local government of central China has not regulated house prices toughly. Even if the housing price regulated by policies exists, the housing price is still rising steadily, especially in the researched capital cities of provinces and the urban centre^53,54^. Meanwhile, there are lags between policy implementation and housing price changes so that the housing restriction policies do not affect the housing price immediately^55^. To evaluate the trend of the historical ACRHP, the Mann-Kendall test^56,57^ was applied at a 0.05 significance level (Fig. 6). The results show that historical ACRHP is in a state of continuously significant increasing. In other words, the actual influence of the policy is smaller than we recognised. For example, it is well-known that the policy of home purchase restrictions has been one of China’s harshest housing market interventions to curb the overheating real estate market by imposing restrictions on purchasing power. Chinese central government has implemented basic purchase restrictions in 40 major cities designated by the Ministry of Housing and Urban-Rural Development since 2011. In Wuhan, a purchase restriction order was issued on February 23, 2011. However, it was only three years that the Wuhan Housing Security and Management Bureau held an internal meeting (September 23, 2014) and then announced the complete cancellation of the purchase restriction^58^. From the trend analysis, we can see that Wuhan housing prices have not been significantly affected by the purchase restriction policy, but have continued to accelerate the rise. The results also show that the market totally become one decisive factor determining market positions the real estate market which is also beyond our expectations about the effect of the home purchase restrictions policy. A more realistic simulation of the impact of the policy for housing prices is not as great as we expected, which is limited.

In all, it is true that the Chinese government’s policy has affected housing prices to a certain extent, such as the monopoly of land supply. However, the land monopoly supply system has not changed during the continuous and rapidly rising housing prices period since 2002. Explaining rapidly changing variables with a relatively invariant variable is an incomplete research idea.

## Conclusions

Taking time-series analysis of individual cities to describe the relationship between night-time light imagery and regional housing price are highly correlation. This is a very encouraging result while considering the utilization of night-time light imagery for estimating and predicting ACRHP in areas where lack timely temporal socio-economic statistics. The quadratic function is considered to be the optimal mining model in most capital cities by regression model analysis. Given the complexity of factors that affect the housing price, our research is based on the city-scale ACRHP to reduce the data noise and simplify the model parameters. We demonstrated that the night-time light imagery has a great potential to mine ACRHP. Besides, predicted ACRHP except for Chengdu and Wuhan which was slight deviation, other cities are within the prediction interval which explained our regression mining model still has important reference significance. Furthermore, the method can be used to enrich application research of night-time light data and provides a new reference point of view to exploring housing prices at the city scale. What’s more, there is a lag between government policy and housing prices. The impact of government policies on housing prices is limited. Based on the time-series analysis of individual cities, the relationship between annually ANLI and annually ACRHP was explored at a city scale. The experimental results show that although the government takes measures to regulate the real estate market, Chinese housing price continues to soar.

In addition, this paper uses the regression mining model of the time series prediction of ANLI to predict future average housing prices to evaluate the rationality of the model. The results prove that the prediction housing prices are mainly the same as the official reported statistics prices, which certifies the rationality of the mining model. Therefore, we conclude that using the ANLI of a city is a feasible method to predict ACRHP. To apply this mining model in a region with a developed economy and a high degree of saturated DN values of light images is a question that needs further study. Next, we will study and analyse the coupling relationship between the ANLI and ACRHP in economically developed regions such as the Yangtze River Delta Economic Zone and the Pearl River Delta Economic Zone in China. In addition, we also consider optimizing ANLI data by fusions of recent NPP-VIIRS data or China’s Luojia-1 data after 2013 and historical DMSP-OLS data before 2013.

All in all, this study has great theoretical significance for the real estate market which not only discovered a new pattern that average night-time light intensity (ANLI) is a fair indicator of average commercial residential housing price (ACRHP), but also established a likelihood function relationship between ANLI and ACRHP. Meanwhile, this study also has great practical significance. The results of this study can provide a useful reference for the public to choose the appropriate cities for employment or settlement and offer a very important and interesting reference point for real estate market investment.

## Table A. The ACRHP of 18 inland provincial capitals in China (2002–2013)

.

**Table:**

City	ACRHP (yuan per square metre)											
	2002	2003	2004	2005	2006	2007	2008	2009	2010	2011	2012	2013
Changchun	2421	2155	2260	2393	2558	3250	3489	4142	5178	6131	5540	6026
Changsha	1802	2040	2039	2314	2644	3305	3288	3648	4418	5862	6101	6292
Chengdu	1975	2096	2452	3224	3646	4276	4857	4925	5937	6717	7288	7197
Chongqing	1556	1596	1766	2135	2269	2723	2785	3442	4281	4734	5080	5569
Guiyang	1643	1949	1802	2169	2373	2902	3149	3762	4410	5070	4846	5025
Harbin	2336	2353	2494	2700	2703	3053	3793	4226	5333	5398	5518	6194
Hefei	1753	2088	2550	3006	3110	3307	3592	4228	5905	6326	6156	6283
Hohhot	1498	1552	1648	2057	2368	2596	2731	3887	4105	4367	5445	5233
Kunming	2276	2233	2474	2640	2903	3108	3750	3807	3660	4715	5745	5795
Lanzhou	1643	1858	2282	2590	2614	2967	3145	3624	4233	4747	5698	5868
Nanchang	1688	2367	2430	2587	3126	3558	3461	3774	4566	5939	6419	7101
Taiyuan	2191	3165	2675	3575	3579	3862	4013	4830	7244	6816	6805	7158
Urumqi	2315	2361	2147	2373	2166	2667	3244	3446	4524	5254	5639	6111
Wuhan	1916	2023	2463	2986	3535	4516	4681	5199	5550	6676	6895	7238
Xi’an	2042	2148	2624	2851	3317	3379	3906	3890	4453	6156	6634	6716
Xining	1464	1644	1725	1877	2022	2421	2900	2900	3328	3646	4718	4628
Yinchuan	2207	2139	2177	2593	2399	2408	2828	3523	3792	4376	4575	4856
Zhengzhou	2027	2045	2099	2638	2888	3574	3928	4298	4957	5696	6253	7162

## Table B. The ACRHP of 18 inland provincial capitals in China (2002–2013) after adjusted by the inflation index

.

**Table:**

City	ACRHP (yuan per square metre)											
	2002	2003	2004	2005	2006	2007	2008	2009	2010	2011	2012	2013
Changchun	2406	2100	2113	2303	2462	3016	3237	4151	4845	5673	5414	5899
Changsha	1791	1988	1907	2227	2544	3067	3050	3656	4134	5424	5962	6159
Chengdu	1963	2043	2292	3103	3508	3969	4506	4935	5555	6215	7122	7045
Chongqing	1547	1555	1651	2055	2183	2527	2584	3449	4005	4380	4964	5451
Guiyang	1633	1900	1685	2087	2283	2693	2921	3770	4126	4691	4736	4919
Harbin	2322	2293	2332	2599	2601	2834	3519	4235	4990	4995	5392	6063
Hefei	1742	2035	2385	2893	2993	3069	3332	4237	5525	5853	6015	6150
Hohhot	1489	1513	1541	1980	2278	2409	2534	3895	3841	4041	5321	5122
Kunming	2262	2176	2313	2541	2794	2885	3479	3815	3424	4363	5614	5672
Lanzhou	1633	1811	2133	2492	2515	2753	2918	3632	3960	4393	5568	5744
Nanchang	1678	2307	2272	2490	3008	3302	3211	3782	4272	5496	6273	6951
Taiyuan	2178	3085	2501	3441	3443	3585	3723	4840	6778	6307	6650	7007
Urumqi	2301	2301	2008	2284	2084	2475	3010	3453	4233	4861	5510	5982
Wuhan	1916	2019	2353	2947	3550	4329	4435	5340	5376	6655	7176	7554
Xi’an	2030	2093	2454	2744	3192	3136	3624	3898	4166	5696	6483	6574
Xining	1455	1602	1613	1807	1946	2247	2690	2906	3114	3374	4611	4530
Yinchuan	2194	2085	2035	2496	2308	2235	2624	3530	3548	4049	4470	4753
Zhengzhou	2015	1993	1962	2539	2779	3317	3644	4307	4638	5271	6110	7011

## References

[1] Wu F, Yeh AG-O (1997). Changing Spatial Distribution and Determinants of Land Development in Chinese Cities in the Transition from a Centrally Planned Economy to a Socialist Market Economy: A Case Study of Guangzhou. Urban Studies.

[2] Gregory, P. R. & Stuart, R. C. Comparing Economic Systems in the Twenty-first Century. (Houghton Mifflin, 2004).

[3] Shaw VN (1997). Urban housing reform in China. Habitat International.

[4] Ren Y, Xiong C, Yuan YF (2012). House price bubbles in China. China Economic Review.

[5] Wen HZ, Bu XQ, Qin ZF (2014). Spatial effect of lake landscape on housing price: A case study of the West Lake in Hangzhou, China. Habitat International.

[6] Wu B, Li RR, Huang B (2014). A geographically and temporally weighted autoregressive model with application to housing prices. International Journal of Geographical Information Science.

[7] Suhaida MS (2011). Housing Affordability: A Conceptual Overview for House Price Index. Procedia Engineering.

[8] Man, J. Y. China’s Housing Reform and Outcomes. (Lincoln Institute of Land Policy, 2011).

[9] Hui ECM, Yue S (2006). Housing Price Bubbles in Hong Kong, Beijing and Shanghai: A Comparative Study. Journal of Real Estate Finance & Economics.

[10] Leung C (2004). Macroeconomics and housing: a review of the literature. Journal of Housing Economics.

[11] Ihlanfeldt KR (2007). The effect of land use regulation on housing and land prices. Journal of Urban Economics.

[12] Glaeser EL, Ward BA (2009). The causes and consequences of land use regulation: Evidence from Greater Boston ☆. Journal of Urban Economics.

[13] Chen J, Guo F, Wu Y (2011). One decade of urban housing reform in China: Urban housing price dynamics and the role of migration and urbanization, 1995–2005. Habitat International.

[14] Saiz A (2007). Immigration and housing rents in American cities ☆. Journal of Urban Economics.

[15] Gonzalez L, Ortega F (2013). Immigration And Housing Booms: Evidence From Spain. Journal of Regional Science.

[16] Li C, Chen G, Luo J, Li S, Ye J (2017). Port economics comprehensive scores for major cities in the Yangtze Valley, China using the DMSP-OLS night-time light imagery. International Journal of Remote Sensing.

[17] Li C (2018). DMSP/OLS night-time light intensity as an innovative indicator of regional sustainable development. International Journal of Remote Sensing.

[18] Li, X. Can night-time light images play a role in evaluating the Syrian Crisis? International Journal of Remote Sensing **35**, 10.1080/01431161.2014.971469 (2014).

[19] Li, X. *et al*. Anisotropic characteristic of artificial light at night -Systematic investigation with VIIRS DNB multi-temporal observations. Remote Sensing of Environment **233**, 10.1016/j.rse.2019.111357 (2019).

[20] Elvidge CD (1997). Relation between satellite observed visible-near infrared emissions, population, economic activity and electric power consumption. International Journal of Remote Sensing.

[21] Doll CNH, Muller JP, Morley JG (2006). Mapping regional economic activity from night-time light satellite imagery. Ecological Economics.

[22] Li, C. *et al*. A likelihood-based spatial statistical transformation model (LBSSTM) of regional economic development using DMSP/OLS time series and nighttime light imagery. *Spatial Statistics* (2017).

[23] Li, C., Chen, G., Luo, J., Li, S. & Ye, J. Port economics comprehensive scores for major cities in the Yangtze Valley, China using the DMSP-OLS night-time light imagery. *International Journal of Remote Sensing*, 1–23 (2017).

[24] Li X, Ge L, Chen X (2013). Detecting Zimbabwe’s Decadal Economic Decline Using Nighttime Light Imagery. Remote Sensing.

[25] Forbes DJ (2013). Multi-scale analysis of the relationship between economic statistics and DMSP-OLS night light images. GIScience remote sensing.

[26] Zhang Q, Seto KC (2011). Mapping urbanization dynamics at regional and global scales using multi-temporal DMSP/OLS nighttime light data. Remote Sensing of Environment.

[27] Liu Z, He C, Zhang Q, Huang Q, Yang Y (2012). Extracting the dynamics of urban expansion in China using DMSP-OLS nighttime light data from 1992 to 2008. Landscape & Urban Planning.

[28] Pandey B, Joshi PK, Seto KC (2013). Monitoring urbanization dynamics in India using DMSP/OLS night time lights and SPOT-VGT data. International Journal of Applied Earth Observation & Geoinformation.

[29] Yu B (2014). Object-based spatial cluster analysis of urban landscape pattern using nighttime light satellite images: a case study of China. International Journal of Geographical Information Science.

[30] Sutton P, Roberts D, Elvidge C, Melj H (1997). A Comparison of Nighttime Satellite Imagery and Population Density for the Continental United States. Photogrammetric Engineering & Remote Sensing.

[31] Lo CP (2001). Modeling the Population of China Using DMSP Operational Linescan System Nighttime Data. Photogrammetric Engineering & Remote Sensing.

[32] Levin N, Duke Y (2012). High spatial resolution night-time light images for demographic and socio-economic studies. Remote Sensing of Environment.

[33] Huang Q, Yang Y, Li Y, Gao B (2016). A Simulation Study on the Urban Population of China Based on Nighttime Light Data Acquired from DMSP/OLS. Sustainability.

[34] li C, Ye J, Li S, Guangping C, Xiong H (2016). Study on radiometric intercalibration methods for DMSP-OLS night-time light imagery. International Journal of Remote Sensing.

[35] Zhang L, Qu G, Wang W (2015). Estimating Land Development Time Lags in China Using DMSP/OLS Nighttime Light Image. Remote Sensing.

[36] Wang L, Fan H, Wang Y (2019). An estimation of housing vacancy rate using NPP-VIIRS night-time light data and OpenStreetMap data. International Journal of Remote Sensing.

[37] Osman, M. Combining multi-source satellite sensor imagery to monitor and forecast land use change in Malaysia, University of Southampton, (2014).

[38] Gardner, R. J. Convex bodies equidecomposable by locally discrete groups of isometries. **32**, 1, 10.1112/s0025579300010780 (1985).

[39] Zhang Q, Schaaf C, Seto KC (2013). The Vegetation Adjusted NTL Urban Index: A new approach to reduce saturation and increase variation in nighttime luminosity. Remote Sensing of Environment.

[40] Letu H (2010). Estimating energy consumption from night-time DMPS/OLS imagery after correcting for saturation effects. International Journal of Remote Sensing.

[41] Ziskin, D., Baugh, K., Feng, C. H., Ghosh, T. & Elvidge, C. Methods Used For the 2006 Radiance Lights. Proceedings of the Asia-Pacific Advanced Network **30** (2010).

[42] Chen BY (2016). Spatiotemporal data model for network time geographic analysis in the era of big data. International Journal of Geographical Information Science.

[43] Holt CC (2004). Forecasting seasonals and trends by exponentially weighted moving averages. International Journal of Forecasting.

[44] Zhao N, Liu Y, Cao G, Samson EL, Zhang J (2017). Forecasting China’s GDP at the pixel level using nighttime lights time series and population images. GIScience & Remote Sensing.

[45] Aiken, L. S., West, S. G. & Reno, R. R. Multiple regression: Testing and interpreting interactions. (Sage, 1991).

[46] Hand, D. J., Smyth, P. & Mannila, H. Principles of data mining. (MIT Press, 2001).

[47] Rousseeuw PJ (1984). Least Median of Squares Regression. Journal of the American Statistical Association.

[48] Rousseeuw PJ, Leroy AM (2005). Robust regression and outlier detection. Technometrics.

[49] Li C, Zheng Y, Wu Y (2017). Recovering missing pixels for Landsat ETM + SLC-off imagery using HJ-1A /1B as auxiliary data. International Journal of Remote Sensing.

[50] Yin L (2019). Real estate advertising campaigns in the context of natural hazards. Disaster Prevention and Management: An International Journal.

[51] Deng G, Gan L, Hernandez MA (2015). Do natural disasters cause an excessive fear of heights? Evidence from the Wenchuan earthquake. Journal of Urban Economics.

[52] Zhang L, Hui EC-m, Wen H (2015). Housing price–volume dynamics under the regulation policy: Difference between Chinese coastal and inland cities. Habitat International.

[53] Zhang H, Wang X (2016). Effectiveness of Macro-regulation Policies on Housing Prices: A Spatial Quantile Regression Approach. Housing, Theory and Society.

[54] Hui ECM, Wang Z (2014). Price anomalies and effectiveness of macro control policies: Evidence from Chinese housing markets. Land Use Policy.

[55] Li L, Zhu D, Hu K (2012). Application of PSR Model to the Effects of Real Estate Regulation Policy on House Price:A Case of Beijing. Resources. Science.

[56] Mann HB (1945). Nonparametric Tests Against Trend. Econometrica.

[57] Kendall, M. G. Rank correlation methods. (Griffin, 1948).

[58] Baidu. Purchase restriction order, https://baike.baidu.com/item/%E9%99%90%E8%B4%AD%E4%BB%A4/4154845?fr=aladdin (2020).

